# Scant evidence for Spearman’s law of diminishing returns in middle childhood

**DOI:** 10.1016/j.paid.2006.08.010

**Published:** 2007-03

**Authors:** Rosalind Arden, Robert Plomin

**Affiliations:** Social, Genetic, and Developmental Psychiatry Centre, Institute of Psychiatry, King’s College London, De Crespigny Park, Box Number P080, London SE5 8AF, UK

**Keywords:** Law of diminishing returns, *g* Gradient, Intelligence

## Abstract

In 1927, Charles Spearman suggested that general cognitive ability, or *g*, might be stronger at the low end of ability. We explored the manifold of *g* across the ability distribution in a large sample (range >800 to >4000 individuals) of British twins assessed longitudinally at 7, 9 and 10 years old using two verbal and two nonverbal tests at each age, thus testing effects of age on the saturation of *g*. We rankit-normalized the test scores, then used a median split on the test with the highest factor-loading. We derived the first principal component from the remaining three tests. We performed each analysis for the whole sample (within age) and also separately by sex. The first principal component explains more variance in *g* in the low ability group at every age and in both sexes separately but the *F* ratio eigenvalues show that, except at age 7 and principally in females, the difference between the low and high ability groups is not significant.

## 1 Introduction

The positive manifold of correlations among scores on diverse cognitive tests was discovered, and named as *g*, by Charles Spearman (Spearman, 1904). Spearman’s *g* is the most well-documented construct in the human behavioral sciences. The reliability of *g* is greater than the reliability of height and weight measured in a doctor’s office (Jensen, 1998, p50), its predictive power leaves rival psychometric constructs in the dust yet, despite a century of research, certain properties of *g* are still unresolved.

One unsettled property pertains to the uniformity, or otherwise, of the strength of *g* across the distribution. Presenting some data collected by Abelson on ‘normal’ and ‘defective’ children, Spearman proposed that the *g* saturation might be stronger at the low end of the ability distribution, stating “the more ‘energy’ a person has available already, the less advantage accrues to his ability from further increments of it.” (Spearman, 1927). Spearman does not offer readers anything in the way of an explanation for this idea. Instead, he offers argument by analogy with a technology of the day: steam engines. Doubling the amount of coal shoveled does not double the speed of the engine. Spearman is tentative about his own metaphor: “possibly there exists further a genuine law of diminishing returns for mental as for material processes” (Spearman, 1927). Here Spearman refers to the economic principle that captures the fact that increasing inputs does not guarantee increased output in the same proportion. On the basis of this halting suggestion, much of the research into the nature of *g* throughout the ability distribution now falls under the banner of ‘Spearman’s Law of Diminishing Returns’ (SLODR). Spearman, who had a fondness for Laws (he indexed 31 of them in one of his books, (Spearman, 1937), but did not name this one, might have been surprised.

The published literature exploring the gradient of *g* along the ability distribution is large; a very comprehensive review of a century of work on this topic lists over a hundred studies (Hartmann & Nyborg, 2006). This large body of work can be divided into two kinds with some overlap between them: those that analyse the gradient in *g* over the ability distribution of similar aged-people, and those that analyse the ability distribution over time. We will discuss the former, ability within age-group, research first.

Two reviews of the ability gradient, published within the last decade, found evidence in support of Spearman’s conjecture (Deary et al., 1996; Jensen, 1998). A key study that included high-school age children found substantial differentiation in the strength of the *g* manifold: the latent factor that emerged from scores on a battery of mental tests, and a separate measure of full–scale IQ (WAIS-R) was up to twice as large in the low ability group as they were in the high ability group (Detterman & Daniel, 1989). There is also evidence that the association between reaction time and intelligence (as measured by Alice Heim 4 score) is stronger among low ability subjects (Der & Deary, 2003).

The Detterman and Daniel study stimulated renewed interest in the topic of SLODR. The basic result, that the *g* manifold is greater among those of low ability, received at least partial support in several subsequent studies involving subjects of various ages (Abad, Colom, Juan-Espinosa, & Garcia, 2003; Deary et al., 1996). Two studies that found some support for SLODR also reported that the *g*-loading of the tests in the battery influenced the outcome: the low–high ability group difference in *g* manifold shrinks among highly *g*-loaded tests relative to less *g*-loaded subtests (Deary et al., 1996; Jensen, 2003).

Some studies have ‘found against’ SLODR (Fogarty & Stankov, 1995). One study sampled two large populations of Danish military draftees (*n* > 25,000 in each sample) (Hartmann & Teasdale, 2004). Another study that found no convincing evidence favouring SLODR explored data on children aged 4–9 (*n* = 574) in a French national standardization sample (Facon, 2004). An exhaustive history of SLODR that evaluated data from hundreds of studies found that the balance of evidence supported the contention that the *g* factor accounts for more variance among those in lower ability groups (Hartmann & Nyborg, 2006).

Spearman wondered whether advancing age might decrease the manifold; although it is unclear why he made this suggestion, since he refers only to work by Cyril Burt showing that teachers’ ratings of students correlated more highly with reasoning test scores in younger children than they did with older children (Spearman, 1927).

Published studies examining the factor structure of intelligence across ages (rather than between ability groups) include subject populations ranging from babies to older adults (89+ years) (Hofstaetter, 1954; Li et al., 2004). Such wide age differences make the studies harder to compare with one another. Four longitudinal studies have been published, but only one study (involving gifted compared with non gifted children (Coyle, 2003), controlled for ability while assessing the size of the manifold (Atkin et al., 1977; Olsson & Bergman, 1977; Osborne & Suddick, 1972; Swineford, 1947). Without this, the studies on SLODR over the life-course become harder to interpret. An exhaustive meta-analytic study of SLODR and its interaction with age, found no support for the hypothesis that the *g* manifold attenuates with age, if ability is controlled (Hartmann & Nyborg, 2006).

Thus the phenomenon – the strength of the *g* manifold throughout the ability distribution – is unresolved. As well as inconsistencies in reports on the phenomenon, a satisfactory explanation remains elusive. We acknowledge the difficulties in developing convincing explanations for things that do not exist, but it would be satisfying to find out, if the size of the *g* manifold *is* influenced by ability, why that is so. A further, natural question to ask, in the light of the well-known heritability of this trait, is whether or not any discovered differentiation is under genetic influence. Education has been invoked as an environmental explanation for the SLODR effect (Abad et al., 2003).

In the current study we test the SLODR hypothesis that phenotypic *g* is greater at the lower end of ability in a longitudinal design, examining each sex separately. This is the only study we know of that tests the gradient in *g* within a cohort that is divided into two ability groups, through early and middle childhood. We have no strong hypothesis about age changes or sex differences.

## 2 Methods

### 2.1 Participants

The Twins Early Development Study (TEDS) recruited families following the birth of twins born in England and Wales in 1994, 1995 and 1996 (Trouton, Spinath, & Plomin, 2002). Nearly 16,000 families were contacted, of whom just over 11,000 agreed to participate. Although there has been some attrition, the population remains fairly representative of the British population as a whole. Only a sub-sample of the 1994 cohort has so far participated at 10 years, so the number of subjects included in this analysis at age 10 is smaller than it is at other ages.

In this study we excluded from analysis all children whose mothers had experienced severe problems in pregnancy and children who had suffered severe medical problems or whose birth weight was less than .47 kg. Other exclusions were: data from children with unknown sex or zygosity; data from families who returned incomplete booklets (information on only one twin, for example); and data returned more than 6 months after a relevant birthday. We also excluded children whose scores on tests (described below) fell more than 3 standard deviations (SDs) below the mean or were more than 3 SDs above the mean, in order to avoid distortion of the results by outliers. We then split the population at each age (*n* = 9632 at age 7, *n* = 6169 at age 9, *n* = 1645 at age 10) into two samples. Individuals within a twin pair were randomly assigned to sample A or B. The two samples are related (sharing half or all their genes) so we do not present them as independent replication samples, but we considered that it would be interesting to see whether similar patterns emerged in both samples.

### 2.2 Measures

#### 2.2.1 Children at age 7

The children were administered four tests of cognitive ability by telephone at age 7; for details see (Harlaar, Spinath, Dale, & Plomin, 2004; Petrill, Rempell, Oliver, & Plomin, 2002). At age 7 the verbal tests consisted of the Similarities test (in what way are milk and water alike?) and the Vocabulary test (what does ‘strenuous’ mean?) of the Wechsler Intelligence Scale for Children (WISC-III-UK; Wechsler, 1992). The non-verbal tests were the Picture Completion test from the WISC-III-UK and Conceptual Grouping from the McCarthy Scales of Children’s Abilities (McCarthy, 1972). Cronbach’s alpha is >.8 on these tests with the exception of Conceptual Grouping (alpha = .66).

#### 2.2.2 Children at age 9

Nine-year old participants took four mental tests administered under parental supervision using booklets that were mailed to the parents (for details see (Arden & Plomin, 2006). Tests included a puzzle test adapted from the Figure Classification test of the Cognitive Abilities Test 3 (Smith, Fernandes, & Strand, 2001), a shapes test also adapted from the CAT3 Figure Analogies test that assesses inductive and deductive reasoning, a general knowledge test adapted from the Wechsler Intelligence Scale for Children, Information Multiple Choice (Kaplan, Fein, Kramer, Delis, & Morris, 1999) and a vocabulary test also adapted from the Wechsler Intelligence Scale for Children.

#### 2.2.3 Children at age 10

Four tests were administered online via the internet to 10-year old participants: two verbal reasoning tests derived from WISC-III-PI Information Multiple-Choice and the WISC-III-PI Vocabulary Multiple-Choice. Two non-verbal tests were also administered; these were adapted from the WISC-III–UK Picture Completion and from Raven’s Standard Progressive Matrices (Kaplan et al., 1999; Raven, Court, & Raven, 1996; Wechsler, 1992).

### 2.3 Data preparation

At each age, we inspected the shape of the distribution of each of the cognitive ability tests. Deviations from normality in skewness and kurtosis were evident in more than half the 12 tests (4 tests at each of 3 ages). We experimented with various cut-off points to slice the distribution into ability groups with closely similar standard deviations, but we found that skewness in the observed data contributed to distortions. We then rankit-normalized the scores. This transformation preserves ordinality but alters the intervals to produce a normal distribution: rankit uses the formula (*r* − 1/2)/*w*, where *w* is the number of observations and *r* is the rank (Bliss, Greenwood, & White, 1956). Since psychometric data are neither ratio-scale nor true interval-scale data, we took the view that the benefit of the method outweighs possible distortions. The usefulness of this method is predicated on the assumption that the true scores are normally distributed despite the observed scores displaying some skewness. The evidence seems to favor normality (in true scores) so we retained the rankit-normalized scores. The correlations between the rankit-normalized test scores and the original standardized scores were over .94 (range .97–.98 at age 7, .94–.99 at age 9 and .97–.99 at age 10), indicating that the rankit transformation does not radically alter the basic data.

### 2.4 Selecting the subgroups

Each of the four tests at each age loaded highly on the first principal component (PC1: .55–.81 at age 7, .68–.77 at age 9 and .71–.76 at age 10). We divided our samples at the mean into low and high ability groups using the most *g*-loaded test as the selection criterion. We did not use the total *g*-factor score to divide the samples because that would have introduced artefactual negative correlations between the tests (see Jensen, 2003, p. 27 for elucidation of this point). Since any test has features that are general as well as features that are test-specific, we reasoned that using the test with the most general component as a selection criterion would yield the most informative results. Scores from the selection criterion test were excluded from the subsequent analysis, so each analysis reports the PC1 derived from the 3 other tests. The most *g*-loaded tests were the vocabulary test at age 7, the puzzle test at age 9 and the vocabulary test at age 10.

### 2.5 Analyses

We derived the PC1 (the *g* manifold) from the 3 tests separately for the low ability group and the high ability group and report the variance explained by PC1. We decided that since our analyses depend on only 3 tests, our method would not be improved by following Kaiser’s rule (Kaiser, 1968) to calculate average *r*. We report the PC1 as a function of ability, age and sex to allow comparisons within and between the two sexes and across the developmental period in our study.

## 3 Results

### 3.1 Low versus high ability groups

**Fig. 1:**
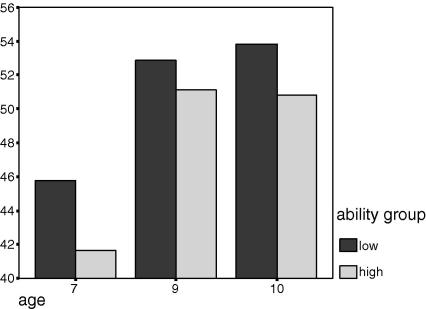
Averaged across both samples: % variance explained by PC1, on the *y*-axis, as a function of age and ability group.

**Fig. 2:**
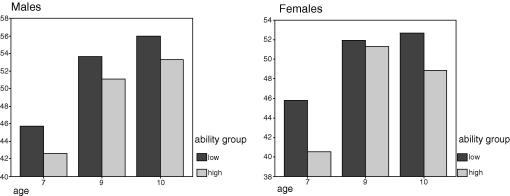
% Variance explained by PC1, on the *y*-axis as a function of age and ability group, split by sex.

**Fig. 3:**
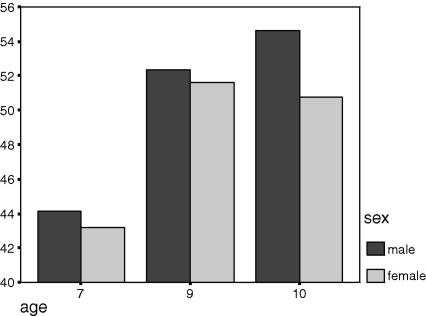
% Variance explained by PC1, on the *y*-axis, as a function of age and sex.

The low groups consistently show a greater manifold (more variance explained by PC1) than do the high groups (Table 1 and Figs. 1–3) at every age in both samples. The only age at which the low/high differences are significant is age 7 and here the difference is carried by the females (*F* = 1.12, *p* = .03) as shown in Table 4 below. The two samples (each sample comprising one randomly selected member of each twin pair) show considerable consistency.

### 3.2 Age effects, boys and girls analyzed together

There is a clear age trend: the size of the manifold increases with age. The PC1 extracted from the 4 test scores (prior to rankit-normalising), after pooling the low and high ability groups, increased linearly (48.45% at age 7, 53.41% at age 9, and 54.31% at age 10). As shown in Table 2, the manifold among the boys increased with age. The girls’ pattern is less consistent though the same general trend is visible.

### 3.3 Within-sex comparisons

We found no significant differences. The *g* manifold of girls in the low ability group is generally higher than the manifold of girls in the high ability group with the exception of girls at age 9 in both samples. Among boys there are no exceptions: the manifold is greater in all low ability groups than the high ability groups. The age effects reported above are also visible when the sexes are analyzed separately. There are some exceptions, but the trend is clear in both samples: the size of the manifold increases with age within both low and high ability girls.

### 3.4 Sex differences

We found no significant differences, although Tables 2 and 3 (see below) show a trend in which the manifold is generally greater in boys than in girls. In both samples, girls at 9 show a greater manifold than boys at 10. This effect is carried by the sub-group of high ability children.

## 4 Discussion

Two clear results emerge from our study: first, the differences we found between the low and high ability groups were, with the exception of the 7 year olds, not significant and second, the low > high effect in the absolute strength of the manifold was systematic despite the differences in tests and administration, across age and across sex. These two results are not as contradictory as they might seem. They might suggest that small differences, latent in middle childhood, will emerge more strongly in adulthood as found in other studies. Another possibility is that the small trend we found will attenuate by adulthood. We found notable sex differences: the manifold is stronger in boys. There is a trend towards the strength of the manifold increasing with age although the effect is somewhat equivocal in girls.

We found that the size of the manifold increased during the small developmental window that we scrutinized; this is the opposite result predicted by Spearman and also goes against the non-significant but discernible trend we found for the *g* manifold to be greater for children of lower ability. How do we reconcile these two apparently contradictory results?

The developmental increase in the size of the phenotypic *g* is what we see when we look, by analogy, down a telescope at a series of populations lined up on an age gradient; older populations have a larger *g* manifold than the younger populations. Continuing the analogy, if we take just one population and put it under a microscope after artificially segregating it into two groups. We see some detail that is not evident in the larger picture. The ‘co-morbidity’ of this polygenic, normally distributed, continuous trait is greater at one end of the distribution than it is at the other end.

It has been suggested that the greater *g* manifold for low ability children is attributable to education (Abad et al., 2003). Our results do not support that conjecture because we find a difference in the size of the manifold between low ability and high ability children who have received a closely similar amount of education, under the standardizing influence of a national curriculum. Our sample is large enough for rather fine differences to be detected and although we clearly see a trend in which the low ability groups show a higher manifold, the differences between groups were not significant.

### 4.1 Limitations

We acknowledge some obvious limitations of our study. Our test battery consists of only four tests. Our results could be specific to these tests, a possibility mentioned elsewhere (Jensen, 2003). Our population is longitudinal: the tests and the test format differ at each age (by telephone at 7, parent supervised, mailed-booklets at 9 and web-based testing at 10). Perhaps the consistency of the result (showing a marginally larger manifold in low versus high ability groups) across different tests and methods of test-administration should strengthen our confidence in the SLODR effect reported by others in older populations.

## 5 Conclusion

Does it matter whether or not the strength of the *g* manifold is uniform? We think it does. Our study showed no evidence of significant differences between low and high ability groups, but because we did find a clear trend in the size of the PC1 we would value knowing whether the small differences will become larger as the children in our samples mature into adulthood. If the nature of *g* differs for the top and bottom halves of the distribution, molecular genetic association research might yield stronger associations in the lower half of the distribution where *g* is reported by others to be stronger.

Since *g* is an important and useful predictor variable it is essential to develop increasingly accurate insights about the strength of *g* along the ability distribution. We hope to have extended usefully the SLODR investigation in two directions – exploring sex differences systematically and inquiring into development through our three-aged study.

## Figures and Tables

**Table 1:** Both sexes: percentage of variance explained by PC1

Age	Ability group	Sample A: PC1	Sample B: PC1
7	Low	45.85	45.72
7	High	42.26	41.04
			
9	Low	53.30	52.42
9	High	50.16	52.04
			
10	Low	54.52	53.15
10	High	50.60	50.98

**Table 2:** Males: percentage of variance explained by PCI

Age & Ability group	Sample A: PC1	Sample B: PC1
7 Low	46.32	45.07
7 High	43.41	41.82
		
9 Low	55.24	52.02
9 High	50.23	51.89
		
10 Low	54.14	57.76
10 High	52.33	54.22

**Table 3:** Females: percentage of variance explained by PCI

Age & Ability group	Sample A: PC1	Sample B: PC1
7 Low	45.61	45.97
7 High	40.87	40.21
		
9 Low	51.88	51.95
9 High	50.27	52.36
		
10 Low	55.91	49.43
10 High	48.86	48.83

**Table 4:** *F* Ratio of Low ability/High ability eigenvalues

Age	Sex	Sample A Low ability PC1 eigenvalue	Sample A High ability PC1 eigenvalue	Sample A *F* ratio (L/H eigenvalues)	Sample B Low ability PC1 eigenvalue	Sample B High ability PC1 eigenvalue	Sample B *F* ratio (L/H eigenvalues)
7	M	1.390	1.302	1.068	1.352	1.254	1.078
		*n* = 1086	*n* = 1218	*p* = .132	*n* = 1098	*n* = 1200	*p* = .101
7	F	1.368	1.226	1.116⁎	1.379	1.206	1.143⁎⁎
		*n* = 1284	*n* = 1136	*p* = .029	*n* = 1249	*n* = 1190	*p* = .010
7	Both sexes	1.376	1.268	1.085⁎	1.371	1.231	1.114⁎⁎
		*n* = 2375	*n* = 2356	*p* = .024	*n* = 2353	*n* = 2394	*p* = .004
9	M	1.657	1.507	1.100	1.591	1.557	1.022
		*n* = 696	*n*=756	*p* = .100	*n* = 698	*n* = 739	*p* = .385
9	F	1.556	1.508	1.032	1.558	1.571	0.992
		*n* = 843	*n* = 820	*p* = .325	*n* = 839	*n* = 818	*p* = .546
9	Both sexes	1.599	1.505	1.062	1.572	1.561	1.007
		*n* = 1539	*n* = 1576	*p* = .118	*n* = 1537	*n* = 1557	*p* = .445
10	M	1.624	1.570	1.034	1.733	1.627	1.065
		*n* = 179	*n* = 167	*p* = .414	*n* = 168	*n* = 187	*p* = .337
10	F	1.677	1.466	1.144	1.483	1.465	1.012
		*n* = 247	*n* = 229	*p* = .151	*n* = 237	*n* = 238	*p* = .463
10	Both sexes	1.636	1.518	1.078	1.595	1.529	1.043
		*n* = 426	*n* = 396	*p* = .224	*n* = 405	*n* = 425	*p* = .334

⁎ *p* < .05.

⁎⁎ *p* < .01.
